# Prevalence and factors associated with antepartum depression among adolescent women in the assin north district of Ghana: a cross-sectional study

**DOI:** 10.1186/s12905-024-03111-1

**Published:** 2024-05-06

**Authors:** Hannah Amoquandoh Asante, Shadrach Tetteh Boyetey, Ebenezer Essaw, Christopher Amoah Nyame, Bertha Mante, Louisa Dziwornu, Paul Okyere

**Affiliations:** 1 Department of Health Promotion and Disability Studies, Kwame Nkrumah University of Science and Technology, Kumasi, Ghana; 2 Department of Health Policy, Management and Economics, Kwame Nkrumah University of Science and Technology, Kumasi, Ghana; 3 Department of Occupational and Environmental Health and Safety, Kwame Nkrumah University of Science and Technology, Kumasi, Ghana; 4 Department of Real Estate and Land Management, University for Development Studies, Tamale, Ghana; 5 Department of Sociology and Social Work, Kwame Nkrumah University of Science and Technology, Kumasi, Ghana

**Keywords:** Antepartum depression, Adolescent, Pregnant women, Ghana, Mental health

## Abstract

**Background:**

Globally, depression is a leading cause of disease-related disability among women. In low-and-middle-income countries (LMICs), the prevalence rate of antepartum depression is estimated to range between 15% and 57% and even higher in adolescent antepartum women. Although a number of studies have shown that depression is common in adolescent pregnancies and has a prevalence rate between 28% and 67% among adolescent mothers, there currently exists no literature on depression among adolescent pregnant women in Ghana. The study aimed to determine the prevalence of antepartum depression and identify the factors associated with it among pregnant adolescent women.

**Methods:**

A quantitative cross-sectional study design was adopted by randomly recruiting 220 adolescent pregnant women visiting antenatal clinics in five selected health facilities in five communities in the Assin North District of Ghana. Data were collected using the Edinburgh Postnatal Depression Scale (EPDS). Data analysis was performed using Stata version 14. Both descriptive and inferential analyses were performed. A chi-square analysis was conducted to identify the association between independent and dependent variables. A multivariate logistic regression analysis was carried out to identify the independent variables that were significantly associated with the dependent variable. In all analyses, p-values ≤ 0.05 were deemed statistically significant at a 95% confidence interval.

**Results:**

The results indicated prevalence of depression was 38.6% using the EPDS cut-off ≥ 13. Respondents who were cohabiting were less likely to experiencing antepartum depression compared to those who were single (AOR = 0.36, 95% CI: 0.20–0.64, *p* = 0.001). Also, Respondents who had completed Junior High School had a lower likelihood of experiencing antepartum depression compared to those who had no formal education (AOR = 0.19, 95% CI: 0.05–0.76, *p* = 0.019). Respondents who perceived pregnancy-related items to be costly had higher odds of experiencing antepartum depression (AOR = 2.05, 95% CI: 1.02–4.12, *p* = 0.042). Lastly, adolescent pregnant women who reported that pregnancy-related items are costly were likely to experience antepartum depression compared to those who did not report such costs (AOR = 2.12, 95% CI: 1.20–3.75, *p* < 0.001).

**Conclusion:**

The results of this study highlight the importance of a multi-pronged strategy for combating antepartum depression in adolescents and improving the overall health and well-being of pregnant adolescents. Considering that adolescence is a transitional period occasioned by several bio-psycho-social challenges, setting up systems to ensure that young girls are motivated and supported to stay in school will enhance their economic prospects and improve their standards of life while providing psycho-social support will benefit their health and general well-being.

## Introduction

Depression is a leading cause of disease-related disability among women globally [1]. Characterized by the Massachusetts General Hospital Comprehensive Clinical Psychiatry as the presence of depressed mood or loss of interest, most of the day, more days than not, depression presents numerous symptoms including changes in appetite and sleep patterns, feelings of guilt, poor self-esteem, poor concentration, psychomotor agitation, fatigue, and even suicide. The prevalence of depression is deeply concerning with an estimated 280 million people living with the disease globally [1]. Among women with a history of pregnancy and/or childbirth, depression in the period before childbirth was common with a prevalence rate as high as 25% [2]. In low-and-middle-income countries (LMICs), the prevalence rate of antepartum depression is estimated to range between 15% and 57% [3] and it is slightly higher than postpartum depression ranging between 14% and 50% [4]. Evidence shows that when not treated, antepartum depression can continue to the postpartum period [5].

Traditionally, pregnancy was perceived to protect women from depression since it was thought to be a period of wellness and happiness but recent evidence suggests otherwise as the incidence of depression diagnoses during childbirth has been on the rise, with a sevenfold increase in 2015 compared to the year 2000 [6]. According to a recent Center for Disease Control(CDC) study, depression during pregnancy is not easily noticeable though its prevalence is high [2]. Evidence suggests it is difficult to diagnose depression during the first trimester because there is usually an overlap between symptoms of pregnancy and symptoms of depression [7]; nonetheless, a systematic review [8] documented a 7.4% prevalence of depression during the first trimester in their study. This figure rose to 12.8% during the second trimester and remained at 12% during the last trimester. Another longitudinal study in Sweden [9], found a 17% rate of antepartum depression. These patients were then followed into the postpartum period and depression rates were found to have decreased by 4–13% by the 8th week after delivery and maintained at 6 months postpartum. Two studies [10, 11] have revealed the risk factors connected with depression during pregnancy; personal and family history of depression, childhood abuse, domestic or partner violence, smoking/substance abuse, single motherhood, inadequate social support, lower educational levels, lack of employment and adolescent pregnancies.

Adolescent pregnancy is widely seen as a public health problem due to its health and social implications. Studies show that 11% of all deliveries worldwide and about 14% of all maternal deaths globally are among adolescents aged between 15 and 19 years old with over 90% of such adolescent births occurring in LMICs [1, 12]. Each year, about 21 million girls aged 15–19 years in low-income and middle-income countries (LMICs) experience pregnancy, with around 12 million of them delivering babies [13]. Ghana’s adolescent pregnancy situation is no different from other LMICs [14]. Ghana continues to record high cases of adolescent pregnancies. Research reveals that about one in ten females between 15 and 19 years of age had begun childbearing in cities whereas the situation in rural settings is twice as high as those in urban settings [15]. The 2014 Ghana Demography and Health Survey (GDHS) report revealed that 14% of adolescents aged 15–19 years had begun childbearing with 11% having had a live birth and 3% pregnant with their first child [16]. Adolescent pregnancies are associated with health, psychosocial and economic consequences that make it a disturbing public health concern [8, 17]. Some studies suggest that adolescent pregnancies have a higher incidence of health-related complications such as serious maternal and neonatal outcomes [17, 18]. These include preterm birth, low birth weight, anemia, Sexually Transmitted Infections (STIs), postpartum hemorrhage, and mental disorders such as depression.

Studies have documented several factors that may contribute to adolescent pregnancy. Some societies put pressure on girls to marry and bear children, especially because there is more value on motherhood and marriage [19]. Girls may also choose to become pregnant when there are limited educational and job opportunities [1, 20]. These may be because of lack of access to contraceptives, knowledge gaps, financial constraints, sexual violence, and lack of autonomy among many other constraints [21].

Women living in developing countries such as Ghana are particularly vulnerable to multiple exposures to depression. Studies in Africa show that rates of depression during pregnancy are generally significantly higher and may have longer durations than those in developed countries [4, 22]. Ghana has over 2.3 million people living with various mental health conditions, yet mental health care remains a challenge, with a 98% treatment gap [23, 24]. Another study in Ghana, which looked at the prevalence and risk factors of postpartum depression among 153 mothers of sick and hospitalized infants in a tertiary hospital, found 32.4% mild, 27.4% moderate, and 9.8% severe depression [4]. A separate study [25], also investigated postpartum depression among HIV-positive women in Ghana and established that 10% of the respondents had symptoms of depression at the time of birth and a further 9% six [26] months after birth. Yet another study by [27] compared three screening instruments for postpartum depression. It was found that 11% of the 160 respondents had scores that represent clinically major depression [16, 28] and these are all studies among adult women who are not adolescents. Although some studies have shown that depression is common in adolescent pregnancies and has between 28% and 67% among adolescent mothers [27, 29], there currently exists no literature on depression among adolescent pregnant women in Ghana. Thus, this study aimed to contribute to the evidence base on antepartum depression among adolescent women in Ghana by placing it in a larger context of prevalence and factors associated with it.

## Methods

### Study design and sampling

This study employed a quantitative cross-sectional approach conducted within health facilities. Participants were recruited through a simple random sampling strategy from five health facilities in the Assin North district of the Central region of Ghana. Overall, 220 pregnant adolescent girls were recruited. The sample size was determined using the Cochran [17] formula for estimating sample size based on 5% level of precision/absolute error and a type 1 error of 5%. The expected proportion of the outcome variable in the population-based on previous studies was 17%.

### Participant recruitment procedures

The research units were chosen using a multistage sampling process. First, five health facilities were selected from the 27 health facilities in the district which were stratified based on the levels of care provided. These five (5) health facilities included one (1) Community-Based Health Planning Services (CHPS), two (2) health centers, one (1) polyclinic and one (1) district hospital. They were selected from five out of the seven sub-districts. One CHPS which represents the largest in the district was chosen to represent the selected level of care in the subdistrict. Also to ensure fair geographical representation, two health centers were chosen in their respective sub-districts. Each health facility was representative of the five sub-districts chosen for the study. The sample size was allotted in tens to each selected health facility using the proportional allocation technique. The district hospital had 80 respondents, polyclinic had 40 respondents, the two health centers had 35 respondents each and the CHPS had 30 respondents. Finally, using the lottery approach, study respondents were randomly selected using the antenatal clinic register. Respondents were then contacted during their antenatal clinic visitations at the five selected health facilities. Follow-up home visitations and phone calls were used to contact respondents who were not present at the facilities. Respondents were included on the basis of being a female, aged (10–19) years, pregnant, living in or around the study area, enrolled in a maternal health program (antenatal care) in a health facility in the study area, willing to participate in the study, and written informed consent was obtained to be a part of the study. Respondents were excluded from the study if they were below 10 years and above 19 years of age, pregnant but had serious health issues that may prevent participation, did not live in or around the study area, and did not give informed consent to be a part of the study. All data collection was conducted face-to-face at the selected health facilities.

### Data collection instrument

The Edinburg Postnatal Depression Scale (EPDS) was administered to serve as a data collection tool since it is one of the most acceptable and internationally recognized standard questionnaires used for studies on depression [30, 31]. One study [32], concluded with preference for the EPDS for measuring depressive symptoms in the peripartum populations. The EPDS is made of ten standardized questions with answers and scores. It asks questions such as “in the past seven days, have you felt happy, been able to laugh and see the funny side of things, looked forward with enjoyment to things, etc? Respondents with a score > 10 were considered to be have probable depression (mild) with 30 being the highest score and showing severe depression. In this study, respondents with scores ≥ 13 were considered to have screened positive for depression as comparable to other studies in Ghana which used the EPDS with such a cutoff point. The data gathering instrument also included questions on respondents’ background characteristics and additional questions about social determinants of health. Data were collected from September to November, 2021. All COVID-19 safety protocols were followed during data collection. This involved the use of face masks by data collectors and maintenance of adequate social distance. There was no physical contact with participants however hand sanitizers were used after signing of consent forms.

#### ***Data Collection Method***

The data collection method involved presenting the questionnaire in English and administering it electronically through a Google Form by trained data collectors. Respondents proficient in English were given the questionnaire to self-administer, while those requiring assistance had the questions read aloud by data collectors.

Additionally, some respondents expressed a preference for the local language (Twi). In such cases, translation and validation of questionnaire items were conducted by data collectors proficient in both English and Twi. These data collectors were responsible for ensuring the accuracy and equivalence of the translations during this process.

Prior to the main data collection, pretests were conducted to validate the translated questionnaire and responses. This validation approach aimed to maintain the integrity and reliability of the data collected in the preferred language of the respondents, ensuring accuracy, clarity, and cultural relevance.

### Data analysis

Data analysis was performed using Stata version 14. Both the descriptive and inferential analyses were performed. A chi-square analysis was conducted to identify the association between independent and dependent variables. A multivariate logistic regression analysis was carried out to identify the independent variables that were significantly associated with the dependent variable. In all analyses, p-values ≤ 0.05 were deemed statistically significant at a 95% confidence interval.

## Results

### Socio-demographic characteristics of study respondents

Table 1 shows the socio-demographic characteristics of study respondents. The total number of respondents that completed the survey questionnaire was 220 adolescent pregnant women. The mean age of study respondents was 17.5 (± 1.6). More than half (*n* = 119/ 54.1%) of the respondents were single. Nearly half (*n* = 103/ 48%) of the respondents were in school with over 56.4% (*n* = 124) of the respondents being in JHS.

**Table 1:** Socio-demographic characteristics of respondents

Variables	Frequency (*N* = 220)	Percentage, % (Range)
**Age (years)**		
14–15	31	14.1
16–17	64	29.1
18–19	125	56.8
Mean (± SD)	17.5 (± 1.6)	(14–19)
**Relationship status**		
Single	114	51.8
Married	8	3.7
Cohabiting	98	44.5
**Level of education**		
JHS	124	56.4
SHS	80	36.4
**Position in class**		
1st to 10th position	76	34.5
11th to 20th position	99	45.0
21st to 30th position	41	18.6
> 30th position	4	1.8
**Occupation**		
Schooling	103	46.8
Apprenticeship	32	14.5
Self-employed	9	4.1
Daily laborer	1	0.5
Unemployed	75	34.1
**Paternal educational level**		
No formal education	54	24.5
Primary	21	9.5
JSS	115	52.3
SSS	30	13.6
**Maternal educational level**		
No formal education	89	40.5
Primary	23	10.5
JSS	89	40.5
SSS	19	8.6
**Paternal occupation**		
Self-employed	190	86.4
Daily laborer	14	6.4
Unemployed	7	3.2
Employed	9	4.1
**Maternal occupation**		
Self-employed	214	97.2
Daily laborer	5	2.3
Unemployed	1	0.5
**Living with**		
Parents	108	49.1
Boyfriend	98	44.5
Husband	7	3.2
Other	7	3.2
**Estimated household monthly income**		
≤ 500	213	96.8
600–1000	7	3.2
**Where do you access healthcare from?**		
Within community	177	80.5
Outside community	43	19.5
**All the healthcare services I need is available**		
Yes	209	95.0
No	11	5.0
**Pregnancy-related items are costly**		
Yes	114	51.8
No	106	48.2
**I have registered with the NHIS scheme**		
Yes	219	99.5
No	1	0.5
**Active registration**		
Yes	216	98.2
No	4	1.8

**Source: Field Survey (2021)**

Nearly half (*n* = 108/ 49.1%) were living with their parents. Most (*n* = 213/ 96.8%) of the respondents were in households with an estimated monthly income of less than or equal to GH₡500.00.

### Prevalence of antepartum depression among adolescent pregnant women

Out of a total of 220 adolescent pregnant women, more than a third (*n* = 85/ 38.6%) experienced antepartum depression (EPDS ≥ 13) while the remaining 135 (61.4%) did not experience it. The prevalence of depression in this study was found to be 38.6%.

### Factors associated with antenatal depression among pregnant adolescent women

#### Socio-demographic/social determinants level factors

Table 2 shows the socio-demographic/social determinants factors associated with antenatal depression among study respondents.Respondent’s relationship status (*p* = 0.002), level of education (*p* = 0.001), maternal level of education (*p* = 0.041), paternal occupation (*p* = 0.009), who you are living with (*p* < 0.001), high cost of pregnancy related items (*p* < 0.001), denial of pregnancy by partner (*p* = 0.023), and denial of responsibility by man’s family (*p* = 0.010).

**Table 2:** Social Determinants/socio-demographic Level Factors associated with Antepartum Depression among Study Respondents

Variables	Antenatal depression		*P* – value
	Yes (EPDS ≥ 13) *n* (%)	No (EPDS < 13) *n* (%)	
**Age (years)**			0.120
14–15	15 (48.4)	16 (51.6)	
16–17	29 (45.3)	35 (54.7)	
18–19	41 (32.8)	84 (67.2)	
**Relationship status**			**0.002**
Single	57 (50.0)	57 (50.0)	
Married	2 (25.0)	6 (75.0)	
Cohabiting	26 (26.5)	72 (73.5)	
**Level of education**			**0.001**
Primary	11 68.8)	5 (31.3)	
JHS	36 (29.0)	88 (71.0)	
SHS	38 (47.5)	42 (52.5)	
**Occupation**			0.130
Schooling	48 (46.6)	55 (53.4)	
Apprenticeship	12 (37.5)	20 (62.5)	
Self-employed	4 (44.4)	5 (55.6)	
Daily laborer	0 (0.0)	1 (100.0)	
Unemployed	21 (28.0)	54 (72.0)	
**Maternal educational level**			**0.041**
No formal education	37 (41.6)	52 (58.4)	
Primary	3 (13.0)	20 (87.0)	
JSS	35 (39.3)	54 (60.7)	
SSS	10 (52.6)	9 (47.4)	
**Paternal occupation**			**0.009**
Self employed	65 (34.2)	125 (65.8)	
Daily laborer	9 (64.3)	5 (35.7)	
Unemployed	5 (71.4)	2 (28.6)	
Employed	6 (66.7)	3 (33.3)	
**Living with**			**< 0.001**
Parents	53 (49.07)	55 (50.9)	
Boyfriend	24 (24.5)	74 (75.5)	
Husband	2 (28.6)	5 (71.4)	
Other	6 (85.7)	1 (14.3)	
Other	6 (85.7)	1 (14.3)	
**Pregnancy-related items are costly**			**< 0.001**
**Yes**	57(50.0)	57(50.0)	
No	28 (26.4)	78 (73.6)	
No	65 (35.3)	119 (64.7)	
**Man’s family deny any responsibility**			**0.010**
Yes	18 (60.0)	12 (40.0)	
No	67 (35.3)	123 (64.7)	
**My partner is very supportive and trying his best**			**< 0.001**
Yes	51 (31.7)	110 (68.3)	
No	34 (57.6)	25 (42.4)	
**Live on insufficient resources so don’t know how we will look after the baby**			**0.042**
Yes	18 (54.6)	15 (45.5)	
No	67 (35.8)	120 (64.2)	
**Peer pressure**			0.540
Yes	9 (45.0)	11 (55.0)	
No	76 (38.0)	124 (62.0)	
**Experience of sexual abuse**			**0.001**
Yes	16 (69.6)	7 (30.4)	
No	69 (35.0)	128 (65.0)	
**Gravidity**			**0.006**
Prima-gravida	74 (44.3)	93 (55.7)	
Multi-gravida	11 (22.5)	42(77.6)	

**Source: Field Survey (2021)**

Other factors which showed statistically significant association with antepartum depression among the study respondents were sexual abuse (*p* = 0.001), gravidity (*p* = 0.006), and planning life independent of partner (*p* = 0.009) were significantly associated with antenatal depression as illustrated in Table 2.

Table 3 shows the multivariate logistic regression analysis of the factors associated with antenatal depression. After adjusting for all the variables, respondents that had JHS education (*p* = 0.019), had a mother with primary level education (*p* = 0.04), and less costly pregnancy-related items (*p* = 0.038) were the most significant factors associated with antenatal depression.

**Table 3:** Logistics Regression Analysis of the Factors Associated with Antepartum Depression among Adolescent Pregnant Women

Variables	Bivariate		Multivariate	
	OR (95%CI)	*P* – value	AOR (95%CI)	*P* – value
**Relationship status**				
Single	1.00			
Married	0.33 (0.06–1.72)	0.190		
Cohabiting	0.36 (0.20–0.64)	0.001		
**Level of education**				
Primary	1.00		1.00	
JHS	0.19 (0.06–0.57)	0.003	0.19 (0.05–0.76)	**0.019**
SHS	0.41 (0.13–1.29)	0.128	0.42 (0.10–1.73)	0.227
**Maternal educational level**				
No formal education	1.00		1.00	
Primary	0.21 (0.06–0.76)	0.018	0.21 (0.05–0.94)	**0.040**
JSS	0.91 (0.50–1.66)	0.760	0.73 (0.34–1.61)	0.441
SSS	1.56 (0.58–4.22)	0.380	0.58 (0.16–2.14)	0.413
**Paternal occupation**				
Self employed	1.00		1.00	
Daily laborer	3.46 (1.11–1075)	0.032	2.92 (0.27–1.61)	0.184
Unemployed	4.81 (0.91–25.46)	0.065	3.14 (0.46–21.22)	0.241
Employed	3.85 (0.93–15.88)	0.063	3.82 (0.75–19.36)	0.105
**Living with**				
Parents	1.00		1.00	
Boyfriend	0.34 (0.19–0.61)	< 0.001	0.66 (0.27–1.61)	0.362
Husband	0.42 (0.08–2.23)	0.306	0.60 (0.09–4.27)	0.613
Other	6.23 (0.73–53.47)	0.096	6.91 (0.68–70.50)	0.103
**Pregnancy-related items are costly**				
Yes	1.00		1.00	
No	0.36 (0.20–0.63)	< 0.001	0.47 (0.24–0.96)	**0.038**
**Sexual abuse**				
Yes	1.00		1.00	
**Gravidity**				
Prima-gravida	1.00		1.00	
Multi-gravida	-	-	-	-
**Denial of pregnancy by partner**				
Yes	1.00			
No	0.52 (0.17–1.60)	0.253		
**Man’s family deny any responsibility**				
Yes	1.00		1.00	
No	0.36 (0.17–0.800	0.012	0.81 (0.28–2.37)	0.698
**My partner is very supportive and trying his best**				
Yes	1.00		1.00	
No	2.93 (1.59–5.42)	0.001	1.31 (0.51–3.36)	0.574
**I will plan an independent life**				
Yes	1.00		1.00	
No	0.46 (0.26–0.83)	0.010	0.60 (0.26–1.42)	0.245
**Live on insufficient resources so don’t know how we will look after the baby**				
Yes	1.00		1.00	
No	0.47 (0.22–0.98)	0.045	0.75 (0.28–1.99)	0.563

**Adjusted for all the significant variables in the multivariate analysis**

**Source: Field Survey (2021)**

## Discussion

### Prevalence of antepartum depression among adolescent pregnant women

The results of this study indicated that 38.6% of the respondents screened positive for depression using the Edinburg Postnatal Depression Scale (EPDS) of cut-off ≥ 13. To the best of our knowledge, this is the first study in Ghana on antepartum depression focusing on adolescent pregnant women. The EPDS has shown a good clinical value and it is compatible with studies conducted in many countries all over the world including Ghana and other SSA countries as a screening tool for depression studies [4, 10]. It is also the most commonly used depression screening tool used for the adolescent population [33].

The study revealed a high proportion (38.6%) of pregnant adolescents with depressive symptoms in the five health facilities in the Assin North District. This is comparable with previous studies [2, 34]. For example, a study conducted in South Africa in a peri-urban area outside of Cape Town reported a prevalence of antepartum depression and Post- Partum Depression (PPD) of 39% and 34.7% respectively [28].

Other studies reported a much higher prevalence of antepartum depression. For example, in a study of rural pregnant women in KwaZulu-Natal, South Africa, 47% of the women showed positive signs of depression, and 45% of those who were depressed were HIV-positive [33]. Uthaipaisanwong et al. [26], recorded 46% whilst a 43.9% prevalence was found by a study conducted on prenatal depression among pregnant adolescents in the Maha Sarakham province [35]. In Ghana, one study found a point prevalence of 69.9.% among mothers with sick infants in Kumasi and currently the highest prevalence of depression in the country [4].

In contrast, there are studies that reported a lower prevalence of antepartum depression. Bindt et al. [8], reported antenatal depression of 26% prevalence, Lillie et al. [36], reported 19.7% whereas Woebong et al. [20], reported 9.9% in studies conducted in Ghana. A study by Birkeland et al. [29], in the United States of America (USA) on adolescent motherhood and depression found a prevalence of 29%. Another study on the association of social support and antepartum depression among pregnant Peruvian women reported a roughly 25%-point prevalence of antepartum depression [20]. Dibaba et al. [4], recorded a point prevalence of 19.9%. In a study conducted in Nepal, depression among women during their antepartum period visiting public health centers was recorded to be 18% [37], while one study in Nigeria reported 16.4% antepartum depression [38] with Gelaye et al. [5], reporting a 10.3% prevalence of antepartum depression in Peru.

The differences in prevalence rate for antepartum depression are influenced by many factors. These factors consist of the study design, the screening tool used and cut-offs, the respondents involved, and the study location. The original EPDS has a minimum cut-off of 10 which indicates signs of mild depression [4]. The revised EPDS guide [39], employs a minimum of 9-11to show mild depression. In this study, the EPDS with a cut of point of 13 and above was employed as the main depression tool. The study was also cross-sectional and conducted in a rural district of Ghana focusing on adolescents. Theses may have explained the relatively high prevalence of depression found in this study since, for example, adolescent mothers are reported to experience a high rate of antepartum depression due to the fact that they are joggling with their identities and responsibilities as adolescents and having to add motherhood to the many challenges they face [40]. Other studies which used the EPDS with cut off of 13 and above include (*P* = 33.1%) [8], (*P* = 32.9%) [34], (*P* = 21.5%) [38]. Studies using the EPDS of 10 and above include Gold et al., (*p* = 37%) [4], and Joshi et al., (*p* = 18%) [36]. Another study used the EPDS of 11 and above and recorded a point prevalence of antepartum depression to be 46% [40]. These are suggestive that perhaps, the EPDS depression scale yields a relatively high depression prevalence.

Conversely, there were studies that utilized other screening tools like the Patient Health Questionnaire (PHQ-9) the Structural Clinical Interview for Depression (SCID) or a combination of SCID and EPDS or PHQ-9 and EPDS. For instance, in Friedman et al. [20] used the PHQ-9 with a cut-off of 15 and above was used to assess the association between social support and antepartum depression among pregnant Peruvian women, reporting a prevalence of nearly 25% [20]. In another study conducted in Ghana and Cote D’Ivoire, the PHQ-9 was used to screen for depressive symptoms yielding a prevalence of 26% and 32% respectively [16]. Rochat et al. [33] used a combination of the EPDS ≥ 13 and the SCID which resulted in a prevalence of 44.5% and 47% correspondingly.

### Association between socio-demographic characteristics and antepartum depression among adolescent pregnant women

The present study found perceived low cost of pregnancy-related items, respondents and maternal levels of education to be significantly associated with adolescent antepartum depression. It revealed that those who perceived the cost of pregnancy-related items to be low were 59% less likely to develop antepartum depression as compared with those who perceived the cost of pregnancy-related items to be high. Though the study did not find poor socio-economic status as a precursor for adolescent antepartum depression, it is understandable that people with poor socio-economic status most likely will find pregnancy-related items costly especially in a rural setting like the Assin North District. Besides, other studies have found associations between poor socio-economic status and antepartum depression [34, 38, 41]. indicated in their findings that poor socioeconomic status and lower-income among pregnant women including adolescents were considerably linked to developing antepartum depression than those with higher income. Being financially dependent and unable to provide their basic needs/care without external support are reasons that can lead to extreme stress and depression among pregnant adolescents. This shows that perhaps, inadequate financial resources may contribute to the concern of not being able to support the financial needs of the upcoming child.

The study also revealed that respondents with JHS education and those whose mothers had primary education were 81% and 79% less likely to be depressed compared to those with primary and no education respectively. Possibly, this is an indication of the importance of formal education on the health and well-being of adolescent girls. Education increase job or economic prospect and those with little or no education are to be at risk of developing antepartum depression [42]. Also, perhaps if the adolescent’s mother is educated, she will understand and give better support to their girl children as love and support are protective factors against the development of adolescent antepartum depression [38]. These results indicate that more attention should be given to adolescents’ health and wellbeing by focusing on ways to improve their educational achievement as well as economic opportunities.

## Conclusions

In conclusion, this study successfully determined that 38.6% of pregnant adolescent women in the Assin North District of Ghana screened positive for antepartum depression using the EPDS with a cut-off of ≥ 13. Factors such as perceived cost of pregnancy-related items, maternal education levels, and socioeconomic status were identified as significant contributors to antepartum depression among this population. These findings highlight the urgent need for targeted interventions aimed at improving access to mental health support, reducing financial barriers to maternal care, and enhancing educational opportunities for pregnant adolescents in Ghana.

Moving forward, tailored strategies focusing on addressing these specific factors are crucial in mitigating the prevalence of antepartum depression and promoting the overall well-being of pregnant adolescent women in the region.

### Limitations

This study has a few limitations. First, the cross-sectional nature of the study precludes any suggestion of causality. Secondly, data were collected in only one district in the Central region of Ghana and the fairly small sample size may not allow for the generalization of the results to all adolescent pregnant women in Ghana. A longitudinal study on the prevalence and associated factors on antepartum depression among pregnant adolescents is thus encouraged.

## Data Availability

The datasets used and/or analyzed during the current study are available from the corresponding author on reasonable request.

## Abbreviations

CHRPE: Committee on Human Research, Publications, and Ethics
EPDS: Edinburgh Postnatal Depression Scale
GDHS: Ghana Demography and Health Survey
LMICs: In low-and-middle-income countries
PHQ-9: Patient Health Questionnaire
PPD: Post- Partum Depression
STIs: Sexually Transmitted Infections
SCID: Structural Clinical Interview for Depression
